# Item

**Published:** 1901-09

**Authors:** 


### ITEM.

### State Medical Examining Board.

THE State Medical Examining and Licensing Board, representing the Medical Society of the State of New York, held a special meeting at Buffalo, August 29-30, and 31, 1901. The sessions were convened at the Kenilworth.

